# The implementation of medical revalidation: an assessment using normalisation process theory

**DOI:** 10.1186/s12913-017-2710-5

**Published:** 2017-11-21

**Authors:** Abigail Tazzyman, Jane Ferguson, Charlotte Hillier, Alan Boyd, John Tredinnick-Rowe, Julian Archer, Sam Regan de Bere, Kieran Walshe

**Affiliations:** 1 Alliance Manchester Business School, The University of Manchester, Booth Street East, Manchester, M13 9SS UK; 2 Plymouth University, Drake Circus, Plymouth, Devon PL4 8AA UK

**Keywords:** Revalidation, Medical regulation, Policy implementation, Normalisation process theory

## Abstract

**Background:**

Medical revalidation is the process by which all licensed doctors are legally required to demonstrate that they are up to date and fit to practise in order to maintain their licence. Revalidation was introduced in the United Kingdom (UK) in 2012, constituting significant change in the regulation of doctors. The governing body, the General Medical Council (GMC), envisages that revalidation will improve patient care and safety. This potential however is, in part, dependent upon how successfully revalidation is embedded into routine practice. The aim of this study was to use Normalisation Process Theory (NPT) to explore issues contributing to or impeding the implementation of revalidation in practice.

**Methods:**

We conducted seventy-one interviews with sixty UK policymakers and senior leaders at different points during the development and implementation of revalidation: in 2011 (*n* = 31), 2013 (*n* = 26) and 2015 (*n* = 14). We selected interviewees using purposeful sampling. NPT was used as a framework to enable systematic analysis across the interview sets.

**Results:**

Initial lack of consensus over revalidation’s purpose, and scepticism about its value, decreased over time as participants recognised the benefits it brought to their practice (coherence category of NPT). Though acceptance increased across time, revalidation was not seen as a legitimate part of their role by all doctors. Key individuals, notably the Responsible Officer (RO), were vital for the successful implementation of revalidation in organisations (cognitive participation category). The ease with which revalidation could be integrated into working practices varied greatly depending on the type of role a doctor held and the organisation they work for and the provision of resources was a significant variable in this (collective action category). Formal evaluation of revalidation in organisations was lacking but informal evaluation was taking place. Revalidation had not yet reached the stage where feedback was being used for improvement (reflexive monitoring category).

**Conclusions:**

Requiring all organisations to use the same revalidation model made revalidation easy to integrate into existing work for some but problematic for others. In order for revalidation to be fully embedded and successful, impeding factors, such as a lack of resources, need to be addressed.

## Background

Revalidation is the process by which ‘all licensed doctors are required to demonstrate on a regular basis that they are up to date and fit to practise in their chosen field and able to provide a good level of care’ [1]. In 2012, following over a decade of debate, medical revalidation was introduced in the UK marking the most significant change in the regulation of doctors in decades [2]. The aim of revalidation, as set out by the profession’s regulator, the General Medical Council (GMC), was to give ‘extra confidence to patients that their doctor is being regularly checked by their employer and the GMC’ and to ensure all doctors meet professional standards [1]. The revalidation framework is based on ‘Good Medical Practice’, [3] the GMC’s core guidance for doctors. The process is usually carried out in a five yearly cycle, the outcome of which is informed by continuing professional development (CPD), annual appraisals, and reflection on the mandatory supporting information (SI) required for these. Table 1 summarises the responsibilities of the majority of doctors, organisations and the GMC in the revalidation process, and the potential outcomes of revalidation.

**Table 1:** Revalidation core model: responsibilities of doctors, organisations and the GMC [1, 28]

Doctors	Organisations/ Designated body	GMC
Licensed doctors who are not trainees must: - Have a connection to one organisation (known as a designated body). - Take part in regular appraisal - Collect supporting information for appraisal: 6 types • CPD (annually) • significant events (annually) • review of complaints and complements (as occur) • quality improvement activity • feedback from colleagues • feedback from patients - Reflect on supporting information All trainees must: - Have a connection to either a Local Education Training Board or Deanery - Supporting information and discussion of progress and learning needs as generated for curriculum and training programme.	Organisations are required by the GMC to provide: - a RO new role, responsible for the revalidation recommendation) - an up to date appraisal system and ensure every licensed doctor has a regular appraisal - a sufficient number of trained appraisers - clinical governance systems that can provide supporting information - policies and systems for identifying and responding to concerns about doctors - link with other organisations where doctors work, so information about their practice can be shared RO makes one of three possible revalidation recommendation to the GMC: - Revalidation - Deferral (request for GMC to provide more time for revalidation decision). Does not affect licence to practise. - Non-engagement (can lose licence)	As the regulator the GMC is responsible for: - setting guidelines - Making the final revalidation decision based on RO’s recommendation (to revalidate; how much time to provide for deferral and whether to refer to a Fitness to Practise Panel) - Provide an Employer Liaison Service to help responsible officers with revalidation and the management of concerns about doctors

Revalidation should be seen in a wider context where a shift has taken place in the public sector away from simply trusting professionals to self-regulate towards an active management model; amidst a growing broader culture of accountability [4]. In active discussion since 1998, it is a policy which has polarised the medical community, proposing substantial regulatory change for the medical profession [5]. Revalidation constitutes a change from reactive and incident led self-regulation, what has been called a ‘laissez faire and paternalistic “gentleman’s club”’, to a proactive approach [2].

The potential for revalidation to impact healthcare is, in part, dependent upon how successfully it is embedded into routine practice [6]. Implementation may not be straightforward, however. The medical profession, because of its history of autonomy, has often been resistant to external regulation and has held a deep rooted cynicism towards management [7–10]. In addition, the small amount of literature that exists on revalidation has found much divergence of opinion over its purpose [5, 11].

Much research has attempted to understand the difficulties in embedding complex new policies and interventions in healthcare systems [12–15]. NPT is a framework that allows for systematic exploration of why some processes lead to a practice becoming successfully (or not) embedded (i.e. normalised) and sustained, by attempting to understand the intervention in relation to the work that people do. NPT has previously been used to explore the implementation of complex interventions such as technology in healthcare, [16] delivering digital health at scale, [17] and care pathways for elderly patients [17]. It is based on the premise that ‘interventions become routinely embedded in their organisational and professional contexts as the result of people working, individually and collectively, to implement them’ [18]. NPT proposes that if participants do not understand, support, or consider an intervention worthwhile, or compatible with their existing working lives, then the likelihood of successfully integres four key analytical domains (outlined in Table 3): coherence (i.e. participants understanding, sense making and value of an intervention); cognitive participation (i.e. commitment and engagement by participants); collective action (i.e. the work participants have to do to make the intervention function) and reflexive monitoring (i.e. the evaluative work people do to assess and understand the ways that a new set of practices affect them and others around them) [16]. These domains are non-linear and interact dynamically to provide a comprehensive explanation of the implementation processes. NPT was designed to be applied flexibly, can be used at one or more points in a qualitative study, has been successfully used beyond its original field and provides a robust theoretical framework to understand the dynamics of implementation [19, 20]. The aim of this study was to understand, through the use of NPT, what issues contributed to, or impeded, the implementation of revalidation in practice and what lessons can be learned.

## Methods

### Design and data collection

We conducted a qualitative investigation of the initial stages of implementing revalidation in the UK, between 2011 and 2015, from the perspective of those who were responsible for its development and early implementation. We conducted seventy-one interviews with sixty UK policymakers and senior leaders at different points during the development and implementation of revalidation: in 2011 (*n* = 31), 2013 (*n* = 26) and 2015 (*n* = 14). The number of interviewees in each of our cohorts was determined, as is appropriate for qualitative research, by the notion of theoretic saturation [21]. We found in 2015 that we reached saturation earlier, in part because of the extent to which issues had already been explored in depth in our earlier cohorts of interviews. The first set of interviews, from 2011, was conducted prior to the introduction of revalidation; the second set in 2013, shortly after the introduction of revalidation, and the final set in 2015, once the process of implementation had been underway for three years [20]. The 2013 interviews in particular formed part of an inclusive stakeholder approach which reached out to employers [22].

The topic guides across all three interview sets focused around three main areas: individual roles and relation to revalidation, understandings of revalidation, its purpose and aims and predictions or experiences of revalidations impact. In addition, the first two interview sets also included a section on the measurement and evaluation of revalidation.

### Participants

The interviews were conducted with policymakers and senior leaders based in the UK, selected using purposeful sampling. The aim was to recruit those who were closely involved in the development of policy and practice of revalidation. The interviewees were key actors in the development and design of revalidation, with some (*n* = 11) being interviewed at two time points and others interviewed once (*n* = 49). Interviewees at each time point differed as our aim was to interview the key actors at each stage, and who this was altered across the time period. In terms of NPT theory these participants could be described as the ‘sense makers’. Though an elite group, these individuals were central to the design of revalidation and the initial phases of implementation. They were also responsible for filtering information, expectations, and understandings of revalidation to the medical profession. They had the relevant knowledge and experience in regards to revalidations development and workings prior to and at each stage investigated and, in addition, a number (50%) were practising clinicians, some of whom were part of the first cohort to be put through revalidation (it was reported that many organisations chose to put senior doctors through the revalidation process first, which these participant’s experiences reflect). The participants’ affiliations to organisations are summarised in Table 2.

**Table 2:** Interviewee organisations

2011	2013	2015
Revalidation Implementation Advisory Board (RIAB) General Medical Council (GMC) Royal Colleges of: Anaesthetists General Practitioners; Pathologists; Revalidation Support Team (RST) Independent sector NHS Employers NHS Confederation NHS Professionals NHS Health Trusts Department of Health Strategic Health Authority Scottish Government British Medical Association (BMA) Independent Doctors Federation National Clinical Assessment Service (NCAS) Court of Appeals UK universities^a^ Revalidation Delivery Board	RIAB GMC Royal Colleges of: Anaesthetists Psychiatrists Academy of Medical Royal Colleges (AoMRC) Independent Sector GP practices Locum agencies UK universities Health Watch and Public Engagement Association (HAPIA) NHS England Wales Deanery BMA Independent health Advisory Service (IHAS) NHS Hospitals Health Education England Patient Liaison Groups	RIAB GMC Royal College of Psychiatrists Care Quality Commission (CQC) RST Independent sector NHS England NHS Employers NHS Clinical Commissioners Health Education England Lay representatives Monitor

^a^Professors of Medicine; General Practice; Health Policy; Medical Education; Surgery

Ethical approval for this study was awarded by the Peninsula College of Medicine & Dentistry research ethics committee (no.12/13–122), Plymouth University Faculty of Health and Human Sciences & PSMD Research Ethics Committee (no. 15/16–486), and by the University of Manchester ethics committee (REC 15028).

### Analysis

Interviews were digitally recorded, transcribed and then imported into Dedoose qualitative data analysis software to assist with the organisation of data [23]. A coding framework was developed using the four domains and sub-domains of NPT by using an adapted version of the NoMAD instrument (outlined in Table 3), which was developed to assess implementation processes (Normalization Measure Development is an instrument designed for assessing the implementation of complex interventions) [6]. The adapted NoMaD instrument was applied to the transcripts by coding evidence of the sub-domains in Dedoose [22]. Following coding, two members of the research team (AT and JF) analysed the data across the three interview stages, using the constant comparative method, [24] in order to understand changes and continuities over time. The inductive method of constant comparison analysis involved searching within individual transcripts, making comparison between transcripts within the same cohort, and comparing transcripts from different cohorts for conceptual similarities and differences. This method was combined with the deductive approach of using the four domains on NPT as a framework for the analysis. This approach was chosen as it allowed NPT to be practically applied to the data while allowing for themes to emerge from the data using the constant comparison method and inductive coding. Independent analysis of the transcripts was undertaken by two researchers (AT and JF). Dedoose was used to enable blind coding and verification of code application to check consistency of analysis. Coding and interpretations were also discussed at regular intervals throughout the analysis phase of the study, during collaborative meetings with all authors, to reduce bias.

**Table 3:** NoMaD instrument as adapted for revalidation

Domains	Sub- domains	Sub-domain questions
Coherence	Differentiation	How does revalidation differ from usual ways of working?
	Communal Specification	Do participants have a shared understanding of the purpose of revalidation?
	Individual Specification	How does revalidation affect the work for participants?
	Internalisation	Can participants see the potential value of revalidation?
Cognitive participation	Initiation	Are there key people who drive the revalidation forward and get others involved?
	Legitimation	Do participants believe that being involved in revalidation is a legitimate part of their role?
	Enrolment	Are participants open to working with others in new ways for the purposed of revalidation?
	Activation	Are participants willing to support revalidation?
Collective action	Interactional workability	Can participants easily integrate revalidation into their existing work?
	Relational integration	Does being involved in revalidation disrupt working relationships? Do participants have confidence in other people’s ability to carry out revalidation?
	Skill set workability	Do participants believe work is assigned to those with appropriate skills to carry out revalidation? Is sufficient training provided to enable participants to enact revalidation?
	Contextual integration	Are sufficient resources available to support revalidation? Do management adequately support revalidation?
Reflexive monitoring	Systemisation	Are participants aware of reports about the effects of the revalidation?
	Communal appraisal	Do participants agree that revalidation is worthwhile?
	Individual appraisal	Do participants value the effects revalidation has on their work?
	Reconfiguration	Is feedback about revalidation used to improve it in the future? Do participants modify how they work with revalidation?

Source: Finch et al. 2015 [18]

## Results

In this section our findings are presented structurally around NPT’s four domains and follow the adapted NoMaD framework presented in Table 3, except that differentiation and individual specification are discussed together as they simultaneously featured in participants’ interview answers. The findings of the constant comparative method are presented in each of the domain sections as they appeared.

### Coherence

Coherence refers to participants’ understanding of an intervention and the sense making work they engage in to establish this. This includes how participants understand an intervention’s purpose, whether this understanding is shared, how they understand the intervention to be different from previous ways of working, perceptions of expected benefits, and the degree to which participants value and accept the intervention.

### Differentiation and individual specification

In order to make sense of the work of implementing and integrating revalidation, participants made comparisons to previous ways of working, specifically the conduct of appraisal, and stated the effect it had had on their work. This form of sense making work is referred to in NPT terms as differentiation and individual specification. Many participants had come to understand revalidation as a formalisation of previously existing practices. In 2011, the formalisation of appraisal featured as something that was necessary and perhaps would be addressed by revalidation.

*For me it is actually really much more about the NHS looking at its processes and systems for ensuring quality and its systems of clinical governance and doing effectively what it said it would a number of years ago, which was good systems in place, good systems of clinical governance in place, appraisal for doctors in place, and really seeing revalidation as a side issue and a by-product of having all those good systems in place.* (Int 12, 2011).

This formalisation was referred to in the later interviews as participants recounted revalidation’s impact. Due to the different impacts perceived, there was disagreement about whether formalisation constituted an improvement. Some participants reported that revalidation had improved the previous appraisal system, as the regulatory requirement of formalised appraisal meant doctors were now obligated to undergo appraisal with a trained appraiser. This was felt to have increased the number of appraisals, and improved the quality and robustness of the process, which previously had been ‘ad hoc’ and informal.

*So we’ve seen a shift of mood that suggests that those people are really having better quality conversations with their appraisers.* (Int 5, 2015).

*I’ve heard about appraisal being described in the past as a sort of a cosy chat and I think it was about formalising that discussion, that opportunity and making sure that there was a serious reflection on things like feedback from colleagues, patients and significant events.* (Int 14, 2015).

Other participants believed the formalisation of appraisal brought about by revalidation was detrimental to openness. The addition of a summative element to the appraisal process (which was described as having previously been only formative in many organisations) was perceived by one participant to further add to this, resulting in safety and wellbeing concerns being less likely to be raised. Participants argued that the potential for remediation curtailed open discussion and deterred doctors from being candid about their concerns and support needs.

*[Prior to] medical revalidation, what we had was … the ability to have a conversation that says I’m concerned about my practice in this area, it’d be helpful if I got some support in making sure that I’m okay in this area’. And I think with medical revalidation there is the slight danger that it might choke off the kind of open conversations that there was before because of the threat that that might lead to a deferral or it might lead to a fitness to practise.* (Int 21, 2013).

While revalidation was understood to have resulted in changes to practice across all three data sets, the comparisons made between prior and post revalidation practice and processes suggest that the nature and degree of change revalidation depended on speciality, organisational type and grade of doctor, and on pre-existing organisational processes. This discussion of change once again focused on appraisal. Organisations where there had previously been minimum engagement with appraisal were described as experiencing the biggest changes as a result of revalidation. Similarly doctors understood to work outside or on the edges of organisational systems were also said to have been impacted on the most.

*One of the impacts of revalidation was, [previously] a lot of the large organisations didn’t have appraisals with their own trust grade doctors, with part time doctors, they didn’t know who was on their books, they had honorary contract doctors. And, I think revalidation tightened up the systems administratively, to actually see who owned and who we didn’t own, and some they did appraisals on…and you’ll see it in the figures, very low appraisal rates [pre-revalidation] for some groups, even in the NHS, such as consultants.* (Int 3, 2015).

The change brought by revalidation and the stage of its implementation was dependant on the prior practices and processes of individual doctors and organisations; however, revalidation itself was seen by the vast majority to improve consistency in appraisals.

*What revalidation brings in the opportunity to do it [appraisal] in a consistent way.* (Int 21, 2011).

Though appraisal was the focus in participants’ comparisons of revalidation to previous ways of working, it was not the only comparison raised in the interviews. Further demonstrating differentiation of revalidation by participants, it was also seen to support better information sharing.

*So strengthening those, let’s call them clinical governance systems, to share that information more widely. And we’re certainly seeing it through other drivers. The whole transparency agenda, in part, is being supported by revalidation but it’s not an explicit link.* (Int 4, 2015).

As well as this, a change from a reactive to proactive approach to fitness to practise was perceived.

*It moves it from reacting to only when there’s a problem, to proactively trying to stop problems occurring, and broadly, improve standards. And that’s why I think it’s, in that sense, a good thing…So it’ll drive them in the right direction in terms of people’s capability, their clinical competence, it’ll drive them in the right direction in terms of probity.* (Int 10, 2015).

### Communal specification

In terms of communal specification, a shared understanding of the purpose of revalidation was not held within or across the three sets of interviews. In 2011, 2013 and 2015 the interviews demonstrated a disparity of opinions regarding the purpose of revalidation, with some participants perceiving the purpose to be regulatory (i.e. to deal with poor performance) while others regarded it as a way to raise standards.

Despite varying degrees of scepticism towards the value of revalidation evident in all three sets of interviews, there was a belief evident throughout all three data sets that some form of change in the regulation of the profession was necessary. In principle the value of revalidation was seen, even if some were sceptical about the process itself. The following quote is illustrative of those who understood revalidation’s purpose to be primarily regulatory:

*If the systems not picking out the poorly performing doctors then the system’s not working. I think that has to be one of the primary purposes of the system is to identify poorly performing doctors and so you can look at the number of poorly performing doctors that are identified as a result of introduction of revalidation.* (Int 3, 2013).

Demonstrating the contrasting perspective, the below quotes are indicative of those who saw revalidation’s purpose as raising standards within the medical profession:

*I think it will raise the standards of doctors significantly and steadily over the years that it’s going on. I think it’s got real potential to make a difference.* (Int 4, 2013).

*I think that it has got that kind of overall quality improvement function, and I’m sort of impressed by that, because I kind of hadn’t really believed that that was going to be the case.* (Int 9, 2015).

### Internalisation

When asked about revalidation (in particular its purpose and impact) most participants’ answers were about appraisal, suggesting that for many in conceptualisation revalidation was equated to appraisal rather than being perceived in its totality of components (see Table 1). This is important for coherence as it suggests a particular framing of revalidation which centres on the appraisal process.

As participants made sense of the value and benefits of revalidation through having to operationalize the policy, their acceptance of it and the value they attributed to the process changed. This is understood in NPT terms as internalisation. In the 2011 interviews, scepticism towards revalidation was at its highest and decreased throughout the four year period being investigated. In 2011, participants noted a mixed reaction to revalidation across the profession. One participant for example suggested that ‘a third of doctors were enthusiastic, a third were sceptical and a third objected to the policy’ (Int 6, 2011). Scepticism was attributed at this stage to a range of causes, notably: a confusion or lack of understanding of what the revalidation process would entail, resentment amongst some doctors at being treated like ‘employees’ rather than professionals, and the belief that revalidation was a ‘hoop jumping’ exercise that would have no impact on improving patient care.

*The bureaucracy. The hoops that you need to jump through. You need for every operation that you do; you need a commentary on it…We are big, grown-up boys. It is that kind of thing. It costs. The more bureaucracy you put round these things, the more it costs. We do not introduce drugs into the NHS if they are not cost-effective. Well there needs to be a cost-effectiveness test to this as well.* (Int 4, 2011).

As time progressed, participants appeared to be less sceptical towards revalidation, with scepticism becoming less evident in the 2015 interviews. Participants in the 2015 interviews perceived that attitudes were changing with an increase in acceptance of revalidation.

*I do think the attitude is changing, I think right at the beginning of revalidation, December 2012 there was quite a lot of hostility to revalidation of the appraisal processes. Over the passage of time I think that that is shifted away and people are saying okay it’s a system, it seems to be working, it’s not going away; we’ve just got to work with it.* (Int 6, 2015).

### Cognitive participation

Cognitive participation is the relational work that people do to support the implementation of a new intervention. This includes having key people to drive it forward and get others involved, the belief by individuals that it is a legitimate part of their role, their degree of openness to working with colleagues in new ways, and willingness to continue to support an intervention.

### Initiation

Initiation, key individuals to drive revalidation forward for successful implementation, was evident in the interview narratives, particularly the latter two sets which occurred when revalidation was underway. In 2011, by comparison this factor was considered far less. The new RO role featured as the role most identified by the participants as vital for revalidation’s success. This, however, tended to be illustrated in the interviews through examples of cases where this key role had not been filled by an individual with the necessary training or willingness to drive the intervention forward or where this individual had not been given the support from other key individuals in the organisation.

*A major trend in some organisations is that some ROs are put in place by the organisation, but the organisation from a Chief Exec and a corporate governance point of view, don’t support the doctor. Hence, the doctor feels isolated, and the company is focused on their business, running the service, making money, and the doctor is bringing to them issues around individual doctors, and they didn’t want to know all that. So, I’ve had two ROs in [area] who’ve resigned from their organisation as the RO, because they said they did not have the support of the Board to do their job properly.* (Int 3, 2015).

### Legitimation

In regard to legitimisation, while participants on the whole accepted that some form of regulation of medical performance was needed and a gradual increase in acceptance of the process occurred, there was a suggestion, particularly in the earlier interviews, that revalidation was not seen by some doctors as a legitimate part of their role. For those holding this perspective, engagement in revalidation was understood as dependent on the mandatory nature of the process rather than on the value doctors placed on it.

*Although everybody is subject to the kind of processes of revalidation and it’s kind of law for everybody now, I think it’s pretty clear that people are only really going to engage when they think they have to.* (Int 1, 2013).

The legitimacy of revalidation as part of doctors’ roles was a factor that was reported as increasing over the four year period, but at no point accepted by all.

*There’s still an element of people who I think are either not participating or are participating in a particular way, or a grudging way, that either they don’t have enough trust at local level or perhaps they don’t like how the appraiser is appointed, how they’ve been trained or what their attitude to you is; all manner of local baggage. That sort of culture does seem to be getting eroded.* (Int 5, 2015).

Older doctors were noted in particular to be resistant to revalidation in comparison to their younger counterparts. The substantial cultural and accountability change brought through revalidation was regarded as the reason for this resistance.

*We find, particularly doctors who are near retirement or doctors who have retired and then returned to practice, they sort of poo-poo revalidation in a way because they’ve never taken it se[riously]…they’ve never had to follow an appraisal system in most of their careers, you know; they’ve never thought that revalidation is required for them, would ever touch them and now it does. It’s a cultural…they’re not used to having to do something that a manager is asking them to do; it’s quite an alien thing for them. That’s quite a challenge for them.* (Int 8, 2015).

### Enrolment

Interview data relating to the subdomain category of enrolment featured strongly in the interviews in all three data sets, though frequently in a negative manner. The interviews highlighted that doctors were not always open to working with others in new ways for the purpose of revalidation. A frequently occurring theme across the three sets of interviews was changing relationships between various groups (such as doctors and their employers and the government and consultants and their junior colleagues) and shifting power dynamics. Some earlier participants for example suggested that revalidation had altered the relationship between the government and the medical profession and was a tool for the government to control the medical profession and that this is one reason why doctors felt resentful.

*The new Labour administration, for them it meant a means of control…they did want to bring doctors down a peg or so, there was this feeling that they are prima donnas and do want they want and so on… They brought in job contracts for consultants and all this sort of stuff, the sort of managerialism of trying to control. That was the approach used: regulation.* (Int 8, 2011).

Others suggest that doctors, particularly consultants and older doctors, see revalidation as interfering with their professional independence. In addition it was reported that senior doctors would be reluctant to have an assessment conducted by doctors more junior to them, or administrative staff, as part of their appraisal process.

The shift of power dynamics identified as being brought by revalidation was also reported as having altered doctors’ relationships to their employers, with doctors being treated more like other employees and less of the freedom previously enjoyed by the profession.

*But in secondary care, I think what you’re seeing is, if you like, the individual consultant becoming less powerful and the employer, probably becoming more powerful. And doctors being treated … they’re treated more like employees than they once were - rightly or wrongly - and that’s the way it’s happening, it seems to me.* (Int 10, 2015).

Hierarchical changes were not the only new ways of working with others that some participants viewed negatively. Changes to whom may act as an individual’s appraiser, specifically the use of appraisers from different specialities to the appraisee, was repeatedly highlighted as problematic. This occurred across all three sets of the interview data.

*And I don’t see how a non-anaesthetist has the knowledge and skills to appraise an anaesthetist and I just think it’s too easy for the appraisee to pull the wool over an appraiser’s eyes if the appraiser doesn’t come from that specialty.* (Int 3, 2013).

There was also a suggestion that doctors may respond in a defensive manner to patient feedback because of the historical power difference between the two groups and an assumed lack of knowledge. A lack of appropriate language to facilitate such discourse was raised as a confounding factor in this form of resistance to patient input.

*If you say to a doctor ‘how d’you feel about patients making a critical comments about your practice?’ they start to be very defensive. And I think one of the real challenges we’ve got is how to develop a language that enables doctors and patients to talk to each other in a much more mature way it would take the relationship from one kind of subservience to one of joint empowerment and that really I think is a major task that you have at the moment.* (Int 12, 2013).

### Activation

Activation, willingness to support revalidation, was reported to be mixed in the interviews across different subsections of the medical profession. While it was evident that doctors were not always open to new ways of working for revalidation and a belief that the medical profession had lost power was highlighted, those interviewed identified, in addition to their own willingness, support for revalidation from those who had taken up the responsible officer role. There was a suggestion that a ‘new professional elite’ was coming into existence through the development of RO networks who were working together in new and positive ways.

*The positive side has been that it’s provided a new network of senior doctors, who are talking to each other … they are emerging as a new professional elite …* (Int 3, 2015).

### Collective action

Collective action is the operational work that people do to enact an intervention. This includes whether an intervention can be easily integrated into individuals existing work, if individuals have confidence in others abilities to use the intervention, the provision of sufficient training to enable staff to implement the intervention and the supply of sufficient resources.

### Interactional workability

The integration of revalidation into existing work, or interactional workability, was raised by many of the participants as problematic or difficult. A lack of time featured in the interviews as a key factor in this difficulty. This was experienced as so for both those undergoing appraisals for revalidation and those conducting them.

*I think there has to be still greater professionalism and the professionalism starts with the trusts attitude to appraisal and you know this has to be something that’s recognised. Primary care you get paid for it, I’m not saying you need to get paid for it, but you need time off to do appraisals properly now that often isn’t the case.* (Int 24, 2013).

*I think it takes an inordinate amount of time to prepare for each year’s appraisal.* (Int 7, 2015).

For doctors who do not fit into a ‘standard model’ of revalidation (standard usually meaning those working in a secondary care NHS organisation, fulltime and in training or a consultant post) the difficulty of integrating revalidation was reported as particularly problematic. Revalidation was understood to be difficult for locums, specialty and associate specialist (SAS) doctors, those working part time, doctors in managerial positions or without clinical contact and those working outside of the NHS. Doctors in these positions were described as likely to struggle gaining feedback because of a lack of or not enough patient/ colleague contact and transient working patterns as well as other SI if outside of organisational systems. They were also reported as likely to have little or no access to CPD or quality improvement activities. Participants reported that these difficulties had led to delays in revalidation recommendations being made.

*So, locum doctors often are not engaged, or haven’t got access within organisations to CPD, they often have to solve it themselves, so if they’re not able to meet the CPD requirements. If you’re a locum, and you’re doing one shift, you possibly aren’t able to get patient feedback.* (Int 3, 2013).

There was also concern that other ‘non-standard’ organisations would struggle to implement revalidation. For example, those practising in rural areas reported a lack of local appraisers with geographical distance causing logistical problems.

*When you’ve got 65 hospitals with 300 doctors scattered all over them where do you get the appraisers from? Which it will come as no surprise that in fact it’s quite easy to find appraisers who are trained and skilled in the bigger centres, but you end up sometimes with the position as we have where you’ve got a hospital with five or six connected doctors – ones that are connected to us – and nobody within 50 miles […] And you end up talking about people driving 50 miles each in different directions and meeting somewhere in the middle to have an appraisal.* (Int 2, 2015).

### Relational integration

Overall the interviews expressed frustration at the ‘one size fits all’ mode of revalidation most notably in the latter two interview sets when revalidation was underway. The interview data also demonstrated that relational integration had, over the period being investigated, been difficult to achieve for revalidation. In addition to the difficulty of integrating revalidation into practice, some participants also noted a lack of confidence and mistrust in other members of their profession in terms of their ability to reliably implement revalidation. This concern was apparent in two main ways. Firstly, there were concerns that colleagues/ other doctors would play the system by choosing ‘soft’ (i.e. those likely to give a positive revalidation recommendation) RO’s if possible (especially locums/ locum agencies). There was also a concern that some doctors do not honestly represent their practice, and instead select sanitised, inauthentic documentation and reflections for revalidation.

*I think good doctors will do it but those who are not good want to bend the system it’s very easy for them.* (Int 25, 2013).

*It’s like school, if you remember school, you always knew who was going to be the hard teacher and the soft marker. So, some of the locum agencies have had external ROs appointed, and when they found the external RO was actually being too rigorous in their requirements. Because, they had doctors who were coming in once a year, flying in from overseas, who they wanted to revalidated, and the RO wouldn’t revalidated them, the locum agencies just dumped the RO and get another one.* (Int 3, 2015).

Participants expressed a lack of confidence in whether the metrics collected for revalidation were a valid measure of standards of medical practice. There was also doubt in the robustness of the system against ‘gaming’ and variability. This concern arose from the fact that appraisers make decision based largely on the information provided to them by appraisees and that likewise, the RO is dependent largely on the information provided by the appraiser. It was suggested that patient feedback, for example, was unrepresentative and ‘tokenistic’ as it only needed to be collected infrequently. Associated with this was the fact that much of the supporting information required for revalidation is reliant on self-report by the doctor with no outside scrutiny or triangulation of data. Some participants felt a consequence of this was that it would be difficult to know whether the appraisal process was impacting practice and raising standards if appraisees were misusing or ‘calibrating’ the system for their own benefit. These participants suspected that doctors would quickly learn to manipulate the system in order to maintain control of the regulation of the profession.

*I think doctors are learning very quickly what the minimum requirements are to complete a portfolio to get through revalidation…Doctors will very quickly learn how to use the system for their own benefit, and calibrate it according to how they want it to be. Because, they have managed to keep external review out, for example, patient feedback has been tokenistic, and it can be once in every five years.* (Int 3, 2015).

### Skill set workability

Connected to the concerns discussed above, factors relating to NPTs skill set workability also featured in the interviews as problematic. The interviews overall gave an impression that participants did not believe that those assigned to carry out and engage in revalidation had the appropriate skills and that this lack was related to insufficient training. Variability was raised specifically, in regards to the quality of appraisals, how supporting information was collected and used, and how the process of revalidation was carried out in the various organisations across the UK. The issue of variability was associated with a lack of training and information for appraisers, doctors and ROs to ensure consistency and a clear understanding of the requirements. Participants reported a lack of guidance on what was required for the appraisal process in terms of how much and what type of supporting documentation was required, resulting in doctors collecting unnecessary information, inconsistent appraisals and a lack of quality assurance.

*The quality of appraisal is hugely variable. I have an excellent one at the moment but it’s hugely variable and the amount of information you have to put varies between appraisals as well.* (Int 7, 2015).

*The quality assurance of the appraisal documentation is lacking, and this is something which I think we really do need to grasp for the future, we need to make sure that appraisers are appropriately managed and calibrated and the supporting information is certainly calibrated so that we’re consistent. And this basically creates a bad reputation for revalidation on the basis that the headlines are, well, Doctor X was revalidated but his practice is in special measures, he’s single handed, he has no concept of what an audit is and what clinical quality improvement looks like.* (Int 13, 2015).

### Contextual integration

As with the previous sub-domain, our findings suggested that in regards to contextual integration revalidation had in many cases been faced with impeding factors. There were some reports in the interviews of a lack of infrastructure and resources provided by organisations to support the implementation of revalidation and its uptake. These accounts ranged from investment to infrastructures to enable to the smooth running of the intervention to a lack of IT, time and staff needed to carry out the work for it. Reported experiences of infrastructure and resource provision varied greatly across the interviews with some participants expressing concern that current IT infrastructure could not adequate support the process.

*The concerns about time involvement are entirely justified. The IT systems in the NHS in all four nations is not up to providing the information that people wish to be able to bring to an appraisal process*. (Int 7, 2011).

### Reflexive monitoring

Reflexive monitoring refers to how participants evaluate an intervention, the collection and use of feedback and reports and how the intervention changes over time. This includes being aware of the impact of an intervention, an agreement that the intervention is worthwhile at an individual and collective level, and feedback about the intervention for future improvement.

### Systemisation

Reports on the effects of revalidation (systemisation in NPT terms) raised by participants in the interviews were predominantly informal, based on personal and colleague discussion and anecdotes. Formal systemisation (i.e. feedback collected or reviews undertaken formally by organisations with the purpose of assessing revalidation) appeared to have not yet been undertaken for the majority, which reflects the infancy of the intervention and also its complexity. Indeed, several participants remarked on the difficulty of assessing the impact of revalidation on performance and within organisations. These reports varied significantly by data set.

### Communal and individual appraisal

Communal and individual appraisal tended to be reported simultaneously by participants as they discussed the impact of revalidation and the corresponding value they placed on it as a result. The behaviour and attitudes of doctors was focused on in this. Reflection was believed to have increased which was identified as a positive and highly valued by participants overall. A decrease in resistance from other doctors more generally towards revalidation was also reported, and in turn an increase in the value placed on revalidation:

*I think the main impact, even at doctor level is cultural, so yeah, probably behaviours and attitudes…my views about it have changed quite a lot and I was quite doubtful that it would change things much and I was also quite irritated, as most doctors are, by the sort of tick boxes stuff, so the process stuff, and even still I find the stuff I have to do for NHS England quite onerous in terms of the paperwork burden. But I can, what I can see now is that having got quite a lot of the nuts and bolts of the process kind of going smoothly around appraisal particularly, I’m actually starting to see the benefits and the doctors that I manage have kind of stopped fighting it.* (Int 9, 2015).

Revalidation was also reported as being valued for its impact on wider governance systems, with a suggestion that as this benefit and value was seen by organisations the intervention was well resourced as a reflection.

*I think some organisations have seen the benefits of revalidation, in terms of how it can support governance, how it can bring together quality improvement, significant events, and it can contribute to patient care in their hospital. And, some have seen that and have resourced the role, and put in place a system, ROs, deputy ROs, revalidation managers.* (Int 3, 2015).

### Reconfiguration

Over the time period under investigation it was evident that the participants were starting to see the value of revalidation and the effect it had had on their and others work. It was apparent that, despite the value being identified in revalidation, it had not yet reached a stage of reconfiguration where feedback from participants (individuals and organisations) in revalidation was able to take effect. There was however, some evidence in the interviews of participants and the organisations they worked in beginning to think about how revalidation could be improved in the future.

*I think employers would say to us that it still feels to them over engineered and they’d like a bit more streamlining. I don’t think that’s going to happen in the first cycle … I think people will be inclined to say, can we just tone this down a bit? Right now I don’t think the employers expect any change any time soon but I think if they were asked that’s what they would say to us.* (Int 5, 2015).

## Discussion

Using NPT as a framework we have identified the key factors contributing to and impeding the implementation of revalidation across the time period under investigation. Understandings of revalidation and the effect it has on practice, consensus of purpose, organisational support, ease of integration and resistance from doctors were the key factors identified and shall be discussed in detail later in this section. We have shown that there has been considerable latitude in how revalidation has been implemented, most likely due to the novelty of the policy, with variability in how revalidation has been understood, the work that has been done to support revalidation and how revalidation has been operationalized. This variability was evident across all three time periods investigated.

### Coherence

Revalidation was frequently conflated with appraisal, reflecting perhaps the fact that appraisal had been used by many organisations as an existing and familiar foundation from which to build revalidation and to develop doctors’ understanding of the process. But this focus on appraisal meant that a more holistic understanding of the revalidation process was often missing from participants’ accounts, which could be detrimental to the reach and embedding of revalidation in organisations. The focus on appraisal also highlighted differences in ways of working brought about by revalidation, in particular formalisation and a shift from formative to summative review. The recognition that revalidation had resulted in some improvements to the appraisal system helped to reduce scepticism, but negative experiences of appraisal tended to be attributed to revalidation. The lack of consensus over the purpose of revalidation may be an emergent property of implementation, reflected in the fact that over the time period under investigation coherence with regard to revalidation developed, and is likely to continue to do so as both doctors and organisations become more familiar with revalidation in practice. Despite the focus on appraisal, in their internalisation of revalidation participants showed a more holistic acceptance of the process. The acceptance was however, expressed towards the principles of revalidation and its purpose, while resistance was reported in regards to the application of the process in practice within organisations because of the difficulties and burden experienced by doctors. Improved clarity and consensus on revalidation’s purpose would likely help to remove some of this resistance by reducing the extra work caused by uncertainty about the process.

### Collective participation

The sense makers of revalidation, such as responsible officers and appraisers, appeared to have committed to the policy and its implementation and were situated as the key individuals driving revalidation forward within their organisations. However, when not supported by adequate resources and infrastructure, their ability to drive implementation was substantially limited. Cultural resistance was identified as an impeding factor to implementation. Hierarchical boundaries and traditions meant not all doctors were willing to work with others for the purposes of revalidation, with resistance from some to appraisers being of a different grade or specialty. Some doctors also felt that revalidation diminished some of their professional status and positioned them as more like other employees. Older doctors were described as being particularly resistant to revalidation and facing the biggest cultural shift. But reports of such resistance did decrease over the time period suggesting that as these cultural changes become normalised, in combination with new doctors more familiar with reflexive practices entering the profession, this particular impeding factor may decrease.

### Collective action

Many of the identified impeding factors to the implementation of revalidation fell under the category of collective action. Difficulty in integrating the work of revalidation into existing work was central to this, with three main factors being cited: the ‘one size fits all’ model, a lack of training, guidance and information on the requirements of revalidation at all levels of the medical profession, and insufficient infrastructure and resources (time/staffing levels especially) to support the intervention. While the latter two factors did decrease over time as organisations and individuals became familiar with the process, the limitations on resources and skill sets were the biggest reported sources of resistance to revalidation and caused the process to be perceived as burdensome by some. These issues could be mitigated by making the process easier (and less time consuming) for doctors and reducing variability in the appraisal process and the collection of supporting information. There were also wider concerns about variability and a lack of robustness in appraisal and revalidation processes within and between organisations. The majority of those interviewed noted that it was not the principle of revalidation that was in contention but rather the difficulty of integrating it into working practices. The removal of obstacles, be it data collection issues or lack of time to carry out the required work, should help reduce this resistance and further help in the normalisation of the intervention.

### Reflexive monitoring

The difficulties noted in part result from the ‘one size fits all’ model of revalidation which contains some implicit assumptions about the nature of organisations and existing organisational systems and processes. If revalidation is to be fully embedded in a heterogeneous range of organisational settings and forms and into the practices of all doctors then this issue needs to be addressed, either through a more flexible revalidation process or through the tailoring of the process to different contexts.

Evidence of reflexive monitoring was limited within the interviews, reflecting the relative novelty of revalidation at the times they were undertaken. Given that revalidation was not introduced until 2012, it is unsurprising that there was virtually no impact reported in the 2011 interviews which predated this. Most discussion and awareness of revalidations impact came in the 2015 data set, which had taken place at the furthest point along the revalidation timeline. Despite this there was evidence that the sense makers of revalidation within organisations were starting to think about how the process of implementation had unfolded, both to highlight problems and to suggest improvements. Revalidation was discussed as a work in progress, needing improvement but now accepted by the majority of doctors. Given the controversial nature of revalidation and the length of time it took from conception through policy debate and development to the commencement of implementation, we might conclude that its introduction has been less problematic and contested than might have been expected. While there was some professional resistance, it was mostly directed at practical difficulties arising as doctors and organisations undertook revalidation for the first time; however, the principle and purpose of revalidation was generally accepted and valued.

### Research implications

We have in this paper investigated the initial implementation of revalidation from the perspective of those responsible for its development and introduction, specifically policy makers and clinical leaders. Further research into the practice of revalidation needs to focus more on the experiences and perspectives of those engaged most directly, particularly doctors as appraisers and appraisees and managers and other clinical professionals involved in enacting revalidation. Nevertheless, our findings complement earlier research into the development of revalidation and some components such as appraisal [2, 5, 10] and provide some of the first direct evidence of its implementation and impact.

Our main theoretical contribution has been to extend the use of NPT to explore the implementation of a broad and complex policy, with wide ranging implications for an entire profession, and the wider healthcare system. Much previous work using NPT in healthcare has addressed the implementation of micro level interventions [25–27]. This expanded application of NPT has highlighted a number of factors which seem to have affected the implementation of revalidation. The four dimensions of the framework (see Table 3) had an intuitive relevance and provided a useful explanatory framework for understanding the implementation of revalidation. There is scope to apply NPT more widely to complex social interventions and policy initiatives at the organisational and system level in future.

### Study limitations

Our analyses are exploratory and based on qualitative interviews conducted with elite individuals in the profession which means these findings may be of limited generalisability in relation to other professionals who are not as directly involved in the initial development and design of revalidation. Furthermore, the perspectives of doctors who do not occupy elite or leadership positions of implementing and delivering revalidation may well differ from the perspectives captured here. Interviews were carried out in the early stages of introducing revalidation; therefore, factors contributing to and impeding the implementation of revalidation are likely to have moved on in the time period since data collection. In addition, other theoretical approaches [25] may have led to different insights and have offered additional explanations of the implementation of revalidation.

## Conclusion

Our findings suggest that, over the five year period between the first and final interviews, revalidation gradually became more embedded and accepted in the medical profession. This related in particular to the normalisation of revalidation, familiarisation with the process and the acknowledgement and experience of benefits brought by the intervention. However, it had not yet become fully embedded and scepticism about the process and reluctance to engage were still apparent. This was most related to practical difficulties and under resourcing of the process, particularly in some organisational settings.
